# Study on the Correlation between Barium Radiography and Pulmonary Infection rate in the Evaluation of Swallowing Function

**DOI:** 10.6061/clinics/2018/e182

**Published:** 2018-06-12

**Authors:** Jie Sun, Zeheng Li

**Affiliations:** Department of Clinical Medicine, Xuzhou Central Hospital, Xuzhou, Jiangsu Province, China, 221009

**Keywords:** Dysphagia, Contrast Agent, Barium Sulfate, Infection Rate

## Abstract

**OBJECTIVES:**

To compare the results respectively obtained from the utilization of 60% barium sulfate suspension and Iohexol as contrast agents for videofluoroscopic swallowing studies and the relationship between the clinical application of the two kinds of contrast agents and the incidence of pneumonia.

**METHODS:**

Sixty cases of stroke patients with dysphagia were selected in rehabilitation department of our hospital, and the gender, age, position of the disease, and stroke nature between groups had no significant difference. Among which, 30 patients who were administered 350 mgI/ml Iohexol, and the other 30 patients with 60% barium sulfate suspension as contrast agent. We performed videofluoroscopic swallowing studies with barium 60% *versus* Iohexol within 1 week after admission and 2 weeks after admission.

**RESULTS:**

After 2 weeks in hospital, the aspiration pneumonia incidence of two groups was statistically significant (*p*<0.05), the pneumonia incidence of Iohexol group was lower than barium sulfate group which might have a impossble relevance with barium aspiration.

**CONCLUSIONS:**

During the videofluoroscopic swallowing study of dysphagia after stroke, barium sulfate can enhance the pneumonia incidence, and Iohexol can be widely applied in videofluoroscopic swallowing study.

## INTRODUCTION

As a common clinical disease, stroke can not only result in hemiplegia and other somatic movement dysfunctions, but also lead to different degrees of dysphagia after stroke (DAS). It has been reported that 1 37%-74% of acute stroke patients had different degrees of dysphagia and 37% of aspiration patients could develop into pneumonia. Videofluoroscopic swallowing (VFSS) study is the most common method to examine swallowing function and has proved to be the early diagnostic basis of dysphagia. At present, the VFSS method is considered as the gold standard for the diagnosis of dysphagia 2. The common method in domestic clinic is to add the right amount of water into barium sulfate powder to make 60% barium sulfate suspension. The barium sulfate granules are big and have some disadvantages, such as insoluble in water, so that they cannot be absorbed by human body and easy precipitation. Since DAS patients may conduct aspiration, the lung infection symptom may be caused or aggravated due to aspiration of barium sulfate in case of conducting VFSS. As a nonionic contrast agent, iohexol has low osmotic pressure, 2 times of plasma osmotic pressure, and can minimize the effects on erythrocytes, vascular endothelial cells and body fluid equilibrium. It is widely used in enhanced CT scanning at present 3. Since it can be absorbed by metabolism and does not result in iatrogenic damage after aspiration, it may be good substitute for DAS patients in case of conducting VFSS.

## METHODS

### Ethics, consent and permissions

Ethical approval was given by the medical ethics committee of The Second Hospital of Hebei Medical University.

### Case collection

Sixty cases of stroke patients hospitalized in the department of rehabilitation medicine of Xuzhou Central Hospital from September 2012 to October 2013 were selected. Inclusion criteria: ① Those who complied with Diagnostic Criteria for Acute Cerebrovascular Diseases revised by National Conference on Cerebrovascular Diseases in 1995 4, verified by skull CT or MRI after cerebral infarction or cerebral hemorrhage; ② class II-IV patients screened by water swallow test; ③ those who had the course of disease within 6 months, occurred stroke for the first time or had previous history of stroke, without dysphagia and other sequelae; ④ those who were 20-65 years old, with consciousness and 15 scores of Glasgow Coma Scale (GCS), had stable vital signs, and could understand and carry out simple instructions, with more than 7 scores of abbreviated mental test scale (AMTS) 5; the vital signs were stable and the examination in X-ray department was allowed by the conditions of patients.

### Preparation of contrast agent

① In barium sulfate group, fluid barium sulfate was 60% barium sulfate suspension prepared by 150 g barium powder +100 ml water; semifluid barium sulfate was a pasty barium sulfate food prepared by 60 ml 60% barium sulfate suspension +1g thickener (konjac glucomannan); pasty barium sulfate was a pasty barium sulfate food prepared by 60 ml 60% barium sulfate suspension +2 g thickener (konjac glucomannan); solid barium sulfate was made by painting semisolid contrast agent, prepared by 60% barium sulfate suspension, on graham bread. ② In iohexol group, fluid iohexol group was 50 ml 350 mgI/ml iohexol; semifluid iohexol was prepared by 50 ml 350 mgI/ml iohexol + 0.75 g thickener (konjac glucomannan); pasty iohexol was prepared by 50 ml 350 mgI/ml iohexol + 1.5 g thickener (konjac glucomannan); solid iohexol was made by painting semisolid contrast agent, prepared by iohexol, on graham bread. Radiography was finished together by trained physicians from rehabilitation department and senior physicians from X-ray department and reached a unanimous conclusion.

### Observation content

Real-time recording was conducted for radiography. The images collected by video camera and radiography machine were analyzed and observed frame by frame through slow playback (24 frames/s). The pharynx transit time was observed and calculated 6, which referred to the time that the head of food ball of contrast agent reached esophageal entrance, namely upper edge of cricopharyngeus muscle, from the cross point of root of tongue and mandible after the startup of swallowing. Aspiration conditions were observed. Aspiration means that the barium sulfate enters into laryngeal vestibule and reaches the underneath of vocal fold and latent inhalation refers to no cough or other clinical unwell symptoms within 1 min after aspiration is found by VFSS 7.

### Diagnostic criteria for aspiration pneumonia 8

1 new cough and expectoration had come out recently or original respiratory disease was exacerbated with purulent sputum, accompanied with or without chest pain; 2 fever; 3 lung consolidation symptoms and (or) moist rale; 4 leukocytes >10×10^9^/L or <4×10^9^/L with or without nucleus shifting to left; 5 flake-like and patch-shaped infiltrated shadow or interstitial changes were showed by chest X-ray examination, accompanied with or without pleural effusion. Any item of above 1-4 items adding item 5 can establish diagnosis, except pulmonary tuberculosis, lung tumor, non-infectious interstitial lung disease, pulmonary edema and pulmonary atelectasis. Whether the patients had aspiration pneumonia before VFSS and after 2 times of VFSS were record according to above standards.

### Statistical treatment

Statistical analysis was conducted by mainly applying SPSS16.0 software. Quantitative data were indicated by mean±standard deviation (*x*^–^±s). Normality test and homogeneity test of variances were conducted for the obtained data. Paired t test was applied for the two eligible groups according to data characteristics. Intra-group comparison applied t test and the comparison of qualitative data was tested by applying Fisher exact probability. *a=*0.05 was used as test level and *p*<0.05 indicated that the difference had statistical significance.

## MATERIALS

PHILIPS-TD digitization multifunction gastroenterography machine (Dutch Philips Electronic N.V), Barium sulfate powder (Tsingtao Dongfeng Chemical Co., Ltd.), Iohexol (Yangtze River Pharmaceutical Group Co., Ltd.), konjacglucomannan (Zhejiang Haili sheng Pharmaceutical Company), FA1004 type electronic balance (Shanghai Balance Instrument Factory), SONY HDR-XR150E digital video (SSGE).

Other things: A number of disposable injectors, a number of disposable paper cups, a number of disposable soup spoons, 75% alcohol, a number of cotton balls, stopwatch and scissors.

## RESULTS

### Barium sulfate group

Thirty cases complied with inclusion criteria, including 17 cases of males and 13 cases of females. Their ages ranged from 30 to 69 years old, with an average of (51.83±9.94) years old. There were 22 cases of cerebral infarction, 8 cases of cerebral hemorrhage, 10 cases of unilateral cerebral hemisphere stroke, 10 cases of bilateral cerebral hemisphere stroke and 10 cases of brainstem stroke. The course of disease of enrollment ranged from 4 to 18 days, with an average of (8.93±3.86) days. There were 12 cases of class II, 11 cases of class III and 7 cases of class IV, according to water swallow test (Table 1).

### Iohexol group

Thirty cases complied with inclusion criteria, including 17 cases of males and 13 cases of females. Their ages ranged from 24 to 65 years old, with an average of (53.80±12.64) years old. There were 20 cases of cerebral infarction, 10 cases of cerebral hemorrhage, 10 cases of unilateral cerebral hemisphere stroke, 10 cases of bilateral cerebral hemisphere stroke and 10 cases of brainstem stroke. The course of disease of enrollment ranged from 4 to 20 days, with an average of (9.30±4.09) days. There were 12 cases of class II, 11 cases of class III and 7 cases of class IV, according to water swallow test (Table 1).

All study objects in the above 2 groups could cooperate to finish all examinations. The 2 groups were compared in general data, such as gender, age, site and type of stroke and course of disease, and the differences had no statistical significance. The dysphagia classes in water swallow test between the two groups were compared and the difference had no statistical significance (Table 1). This result demonstrated that the general data of study subjects between the 2 groups had good comparability.

### Comparison of incidence rates of aspiration pneumonia between the two groups within 2 weeks after VFSS

Incidence of aspiration pneumonia of patients in the two groups before VFSS included: 5 in 30 cases of patients in barium sulfate group were diagnosed as pneumonia and 3 in 30 cases of patients in iohexol group were diagnosed as pneumonia, with the total incidence rate of pneumonia of 13.3%. Tested by Fisher exact probability of statistics, *p*>0.05, and the difference in incidence rate of pneumonia between the two groups had no statistical significance (Table 2).

Patients in the two groups were conducted by VFSS within 1 week after admission, and then they conducted swallowing training for 2 weeks subsequently and they were given VFSS again. After 2 times of VFSS, the incidence of aspiration pneumonia of patients in the two groups included 13 cases of patients in barium sulfate group were diagnosed as pneumonia and 5 cases of patients in iohexol group were diagnosed as pneumonia, with the total incidence rate of pneumonia of 30.0%. Tested by Fisher exact probability of statistics, *p>0.05,* and the difference in incidence rate of pneumonia between the two groups had no statistical significance. The incidence rate of aspiration pneumonia of iohexol group was lower than that of barium sulfate group (Table 2).

## DISCUSSION

Dysphagia is common and possibly get more serious complication after stroke. For some patients, it is the only symptom or prominent symptom. The generation mechanism of dysphagia after stroke has been proved to be related with the following sites, including cortex, cortex downward projection, cerebellar and extrapyramidal abnormalities, sensory deprivation and loss of cranial nerve related to swallowing. The central site of cortex swallowing refers to the anterolateral cortex or the outer side of motor cortex, or the area representing front caudal area on primary motor cortex face, or the lowest site of precentral gyrus and the posterior of inferior frontal gyrus 9. Dysphagia not only happens to brainstem and bilateral cerebral hemispheres, but also appears in unilateral hemisphere stroke. Basal ganglia, hypothalamus and amygdaloid nucleus take part in the regulation of swallowing reflex. Stroke leads to myasthenia of tongue, lip, cheek and pharynx, so food cannot be well chewed and stirred to become food ball and be sent to pharynx. Due to paralysis of soft palate and weakly covered back end, food or fluid enter into aditus laryngis to cause severe bucking, thus to cause post-stroke aspiration pneumonia. Foreign literatures reported that about 37%-74% patients with acute stroke had different degrees of dysphagia, the incidence rate of pneumonia for DAS patients could increase by 3.17 times, and the incidence rate of aspiration could increase by 11.5 times. About 43%-54% of DAS patients had aspiration. 37% of such patients developed into pneumonia 10. Dysphagia is the independent risk factor that affects the death and poor prognosis of stroke patients. VFSS is the most commonly used method to evaluate the swallowing function of oropharynx, the ideal method for the evaluation of dysphagia and the gold standard for early diagnosis of dysphagia, serving as important basis for early detection of pharyngeal disorders and their severity. We applied x-ray dynamic imaging video tape recorder and video camera to record its activities simultaneously, then used software to slowly playback frame by frame and conduct quantitative analysis to find the abnormalities. In this study, we chose 60% barium sulfate suspension and iohexol, nonionic contrast agent, to conduct video fluoroscophic swallowing study for DAS patients respectively. Both of them could be used for radiography of mouth, pharyngeal cavity and esophagus. In this study, pharynx transit time was used as evaluation index. Studies showed that about 45% of normal population happened aspiration in deep sleep, but it could not trigger aspiration pneumonia. It is speculated that the incidence of aspiration pneumonia for DAS patients might be related to bilateral or unilateral guttural sensory disorder and weakened pharyngeal reflex caused after stroke. Therefore, lower respiratory infection of DAS patients is correlated with guttural sensory disorder and weakened pharyngeal reflex 11. Pulmonary infection is the most common complication of acute stroke, with the incidence rate of about 7%-21% 12, and is one of the main reasons for deterioration of diseases and death of stroke patients. The aspiration pneumonia caused by post-stroke dysphagia is one of the main reasons for the death of stroke patients.

Chest radiography showed that the wide and high-density shadow in the lungs supports excessive inhalation of barium sulfate 13. Barium sulfate enters into trachea to reach lungs, thus to directly affect respiratory function. Meanwhile, most patients with dysphagia after stroke are accompanied with cough weakness. Sputum and the inhaled barium sulfate cannot be expectorated completely. The barium sulfate granules, which are not expectorated, deposit in lungs and directly generate chemical stimulation and blocking to trachea, thus to cause airway hyperresponsiveness. In early stage, it can cause infiltration of foreign body giant cells, epithelioid cells and monocytes. Subsequently, fibrosis happens around the deposited barium salt to form barium node to result in impaired lung functions, increase of complications, including pneumonia, and obviously increased pulmonary infection rate, thus to cause iatrogenic damage. In this study, the incidence of aspiration pneumonia in the two groups of patients before being conducted VFSS were compared and the difference had no statistical significance. Patients in the two groups were conducted by VFSS within 1 week after admission, and then they conducted swallowing training for 2 weeks subsequently and they were given VFSS again. After 2 times of VFSS, the difference in incidence of aspiration pneumonia of the two groups had statistical significance. Therefore, the incidence rate of aspiration pneumonia of iohexol group is significantly lower than that of barium sulfate group, which may because the utilization of iohexol in VFSS for patients with dysphagia after stroke can avoid the aspiration of barium sulfate, not easily trigger pneumonia and reduce iatrogenic damage.

## AUTHOR CONTRIBUTIONS

Li Z was the guarantor of integrity of the entire study, and responsible for the study concepts, study design and approval of the final version of the manuscript. Sun J was responsible for literature research, clinical studies, experimental studies, data acquisition, data analysis/interpretation, statistical analysis, manuscript preparation, manuscript definition of intellectual content, and manuscript editing.

## Figures and Tables

**Table 1:** Comparison of general data of objects in the 2 groups.

Item	Barium sulfate	Iohexol group	t/*χ*^2^	*p*
Age (*X̅*±s, years old)	51.83±9.94	53.80±12.64	t=1.525	0.506
Gender (male/female)	17/13	17/13	*χ*2=0.0	1.0
Stroke site (unilateral cerebral hemisphere/bilateral cerebral hemisphere stroke/brainstem)	10/10/10	10/10/10	*χ*2=0	1.0
Stroke type (cerebral infarction/cerebral hemorrhage)	22/8	20/10	*χ*2=1.226	0.581
Course of disease of enrollment (*X̅*±s, days)	8.93±3.86	9.30±4.09	t=-0.196	0.722
Water swallow test (II/III/IV)	11/12/7	12/11/7	*χ*2=0.087	0.957

Note: The general data of study subjects between the 2 groups had comparability, *p*>0.05.

**Table 2:** Incidence of aspiration pneumonia of patients in two groups.

Group	Incidence rate of aspiration pneumonia	
	Before VFSS	After 2 times of VFSS
Barium sulfate group (n=30)	5/25	13/17
Iohexol group (n=30)	3/27	5/25
*p-*value	>0.05	<0.05
